# Poster Session I – Poster of Distinction - A93 THE LONGER THE WAIT, THE GREATER THE RISK: A META-ANALYSIS OF SURVEILLANCE INTERVALS FOR PATIENTS AFTER RESECTION OF 1-2 SMALL NON-ADVANCED ADENOMAS

**DOI:** 10.1093/jcag/gwaf042.093

**Published:** 2026-02-13

**Authors:** N Chang, H AlAwadhi, G Leontiadis, N Forbes, Y Yuan, F Tse

**Affiliations:** Gastroenterology, McMaster University, Hamilton, ON, Canada; McMaster University, Hamilton, ON, Canada; Gastroenterology, McMaster University, Hamilton, ON, Canada; University of Calgary, Calgary, AB, Canada; Gastroenterology, McMaster University, Hamilton, ON, Canada; Gastroenterology, McMaster University, Hamilton, ON, Canada

## Abstract

**Background:**

The optimal post-polypectomy surveillance interval for patients with 1-2 small (<10 mm) non-advanced adenomas (NAAs) is uncertain. Previous guidelines were informed by systematic reviews and meta-analyses (SRMAs) that used inappropriate comparator populations (polyp-free individuals or general population), rather than directly comparing surveillance intervals within the same at-risk population.

**Aims:**

This SRMA is the first to compare different surveillance intervals in adults with a baseline finding of 1-2 small NAAs.

**Methods:**

This SRMA was conducted to inform the CAG post-polypectomy surveillance guideline. We searched MEDLINE, EMBASE, and Cochrane Central Register of Controlled Trials up to March 2025 for randomized controlled trials (RCTs) and cohort studies comparing surveillance intervals. Pre-defined comparisons included: <3 vs. 4–5 years, <5 vs. 6–10 years, <6 vs. 7–10 years, <10 vs. >10 years. Two reviewers independently performed study selection, data extraction, and quality assessment. Primary outcomes were CRC incidence and CRC mortality. Secondary outcomes included CRC stage, adverse events, all-cause mortality, and advanced adenoma (AA) incidence. Pooled risk ratios (RR) with 95% confidence intervals (CI) were calculated with a random-effects model. Heterogeneity was assessed by Chi^2^ (P < 0.15) and I^2^ tests (>25%). We assessed the certainty of evidence (CoE) with the GRADE approach.

**Results:**

From 3086 citations, 9 cohort studies (8 retrospective, 1 prospective) were included. No RCTs were identified. Analysis revealed a dose-dependent relationship between longer surveillance intervals and higher risk both within and between studies. Surveillance at <3 years compared to 4-5 years showed no significant difference in CRC risk (RR 1.06, 95%CI 0.69-1.63). Surveillance at <5 years compared to 6-10 years showed a significantly lower risk of CRC (RR 0.37, 95%CI 0.25-0.54) and AA (RR 0.60, 95%CI 0.53-0.69). This protective effect strengthened as comparator intervals lengthened, with the most pronounced reduction for <10 years versus >10 years for both CRC (RR 0.19, 95%CI 0.12-0.30) and AA (RR 0.53, 95%CI 0.37-0.75). The CoE for all outcomes was very low due to serious risk of bias and serious indirectness.

**Conclusions:**

For patients with 1-2 NAAs, longer surveillance intervals are associated with a dose-dependent increase in CRC and AA risks. These findings argue against de-escalating to average-risk FIT screening and support continued surveillance with colonoscopy.

**Funding Agencies:**

CPAC (Canadian Partnership Against Cancer)

